# Views on antioxidant mouthwashes as adjunct in periodontal therapy

**DOI:** 10.6026/973206300161069

**Published:** 2020-12-31

**Authors:** Karthikeyan Murthy Kumar, Sheeja S Varghese

**Affiliations:** 1 Saveetha Dental College, Saveetha Institute of Medical and Technical Sciences, Saveetha University, Chennai 77, India

**Keywords:** Antioxidant mouthwashes, dental plaque, chlorhexidine, gingivitis, periodontitis

## Abstract

Clinical decision is often difficult with chlorhexidine mouthwash. The use of antioxidant mouthwashes for the treatment of periodontal disease is in practise. Therefore, it is of interest to collect gleaned information on Antioxidant mouthwashes as periodontal therapy from known literature. Improvement in treatment using antioxidant mouthwashes is reported in several studies. The mouthwash with antioxidants has similar anti-gingivitis, antiplaque and antimicrobial effects as that of chlorhexidine mouthwash.

## Background

Periodontitis is a chronic inflammatory condition which is multifactorial in nature that results in destruction of the supporting structures of the tooth thereby resulting in the loosening of the tooth and also increases the systemic inflammatory burden thereby influencing various systemic diseases [1,2]. Gram-negative bacteria are considered to be the primary pathogen involved in periodontal destruction, Recent studies have shown that viruses like cytomegalovirus and Epistein-Barr virus contribute to the etiopathogenesis of chronic periodontitis [3]. Periodontitis is a host mediated inflammatory process where there will be elevated levels of cytokines and other inflammatory mediators [4-7]. The genetic plays a role in determining the host susceptibility to periodontal destruction [8]. Periodontitis treatment modalities involve non-surgical and surgical therapy. Nonsurgical treatment modalities involve mechanical and chemical plaque control measures [9-11]. Whereas surgical periodontal therapy involves resective and regenerative procedures [12-14]. Periodontitis when left untreated might lead to loss of teeth, function and aesthetics [15,16]. Replacement of lost teeth can be done by dental implants, which have become a crucial part of prosthetic rehabilitation in periodontitis patients over the recent years [17] and this also requires regular maintenance. However, the fact that significant proportions of individuals fail to routinely perform an adequate level of mechanical plaque removal due to compliance, manual dexterity etc., justifies the implementation of adjunctive chemical aids to enhance the control of biofilms [18-21]. Of the antimicrobial mouth rinses, chlorhexidine is regarded as the gold standard for the prevention of dental plaque [20, 21]. However, it has not been recommended for long-term use because of its reported side effects [22-25]. Hiora mouthwash was recently found to have better antiplaque effects in treatment of gingival conditions [26]. The excessive presence of free radicals caused by oxidative stress or antioxidant deficiency has been linked to periodontal disease [27]. Early in the progression of periodontal diseases, there is a remarkable oxidative process with increased levels of reactive oxygen and nitrogen species. This process can lead to an imbalance in the body response, with changes in biomolecule, resulting in periodontal tissue damage [28]. The antioxidant defense system can reduce the damage caused by reactive oxygen or nitrogen species [29]. Antioxidant mouthwashes have shown to beneficial effect on gingival inflammation helping to reduce the amount of plaque accumulation and subgingival periodontopathic microorganism. Thus, an increasing number of people around the world are turning to antioxidant mouthwashes for both prophylaxis and treatment of different diseases. Literature evidence showed showed that chicory leaf extract, a potent antioxidant when used with nonsurgical periodontal therapy might be helpful in controlling periodontal status [30]. Studies have stated that lycopene, an antioxidant, is a promising treatment modality as an adjunct to full-mouth SRP of the oral cavity in patients with moderate periodontal disease [31]. Recent evidences state that green intake as a component of nonsurgical periodontal therapy is promising for superior and rapid resolution of the disease process [32]. Based on clinical trials, its suggestive that the use of antioxidants systemically act as good adjuvants in periodontal therapy, modulating oxidative stress on the periodontium during periodontitis. Therefore, antioxidant therapy may lead to the maintenance of periodontal health and decrease of inflammatory levels, such as improvement of PI, GI, BOP, and CAL. In the search for adjunct to conventional mouthwashes, studies have shown that antioxidant mouthwashes especially triphala & green tea extract can be beneficial in reducing the gingival inflammation, reduce the plaque accumulation thereby helping to reduce periodontal damage and its systemic effects when compared to antimicrobial mouth rinses that can cause resistance and certain side effects [33-36], whereas few study showed that there is no significant difference between triphala and chlorhexidine in relation to antiplaque and antigingivitis activity [37]. The evidence regarding the supporting role of antioxidant agents as mouthwashes in periodontal treatment is limited which makes clinical decision-making difficult. Therefore, this systematic review is aimed at whether antioxidants mouthwashes have some beneficial effect on the treatment of periodontitis.

## Materials and methods:

The systematic review was carried out according to the PRISMA (Preferred Reporting Items for Systematic Reviews and Meta-Analyses) with the following research question: "Is antioxidant mouthwashes effective in treating periodontal disease?". The institutional review board of the University approved the study protocol. The PIO (population/problem, intervention and outcome) strategy was applied for the systematic article search. All the keywords related to gingivitis and periodontitis from Mesh were used for population/ problem search, the keywords such as antioxidants, and names of various individual antioxidant agents were used for searching the intervention category and keywords pertaining to clinical parameters such as plaque index, gingival index, bleeding index and microbial parameters such as colony forming units were used for the outcome search. Searches were combined with Boolean operators and the articles were downloaded. Two reviewers independently screened articles for eligibility. Studies were considered eligible for systematic review if it was randomized controlled trials on antioxidant mouthwashes comparing its antiplaque and antigingivitis activities with placebo/gold standard chlorhexidine mouthwash and also clinical trials on antioxidant mouthwashes comparing its antiplaque and antigingivitis activities with placebo/gold standard chlorhexidine mouthwash. Studies published in English and studies that reported either plaque index or gingival index or both were included. Reviews, case reports, animal studies, in vitro studies, abstracts, editorials, letters, and historical reviews were excluded. The search was carried out in MEDLINE via PubMed, Cochrane Central Register of Controlled Trials CENTRAL and Google Scholar. All cross-references lists of the selected studies were screened for studies that could meet the defined eligibility criteria. The last date of search was February 29, 2020. The obtained records were subjected to removal of duplicates and then their titles and abstracts were screened for eligibility. As a second, full text papers were obtained if they fulfilled the above mentioned eligibility criteria. Studies were excluded if they did not fulfill all the inclusion criteria. The results of systematic review were presented as a narrative synthesis. 

## Data analysis:

Due to the heterogeneity of the interventions an assessment of outcome, pooling of data was not possible. Due to this Meta analysis was not done instead qualitative synthesis is prepared.

## Caveats on gleaned data:

Quality assessment of selected studies was carried out based on CONSORT Guidelines 4 major criteria to evaluate risk of bias (1) randomization, (2) allocation concealment, (3) Assessor blinding and (4) dropouts. It was considered low risk if 3 out of 4 criteria was satisfied, moderate risk if only 2 criteria was satisfied and high risk if none of the criteria was satisfied.

## Results:

Figure 1(see PDF) gives the number of studies evaluated, screened, assessed for eligibility, and included in the systematic review with reasons for exclusion at each stage. The initial search in the electronic database yielded 74 articles out of which 27 articles were from Google Scholar, 29 articles were from PubMed and 18 articles were from Cochrane. Two authors independently screened titles, abstracts and full manuscripts according to selection criteria. 7 duplicate articles were removed, after reading the title/abstracts 52 articles were excluded. One article was excluded during the full text screen or during data extraction based on the inclusion/ exclusion criteria and finally 14 articles were included in this present systematic review. The characteristics of the included articles were as shown in (Table 1 - see PDF). The final 14 studies compared antioxidant mouthwashes i.e., Triphala, Green tea extract, Neem, Turmeric, Guava leaf extract, Polyherbal (Zingiber officinale, Rosmarinus officinalis and Calendula officinalis), aloe vera and tea tree oil to either placebo, Chlorhexidine mouthwash or Mint mouthwash. In 14 studies, all studies assessed the clinical parameters and 4 studies evaluated the microbiological parameters. 13 studies showed that the antioxidant mouthwashes had improvement in regards to plaque and gingival score when compared to the baseline values, whereas one study did not compare with the baseline value. In 14 studies included, 12 studies compared the antioxidant mouthwashes with chlorhexidine in which 1 study showed that neem antioxidant mouthwash had better effect compared to chlorhexidine, whereas the other 11 studies showed that antioxidant mouthwashes had similar antiplaque and anti-gingivitis as that of chlorhexidine. In 14 studies, 4 studies evaluated the microbiological parameters and showed that both chlorhexidine and antioxidant mouthwashes had similar effects but the chlorhexidine had better substantivity. 4 studies of triphala, 2 studies of green tea, 2 studies of neem and 4 studies on other antioxidants where compared with that of chlorhexidine mouthwash, except for one study on neem showed better antiplaque and anti-gingivitis effect compared to that of chlorhexidine mouthwash, whereas all the other studies had equal effect as that of the chlorhexidine mouthwash. In the 4 studies on triphala, the study assessed the efficacy of triphala mouthrinse on dental plaque and gingivitis in children, the results showed that both chlorhexidine and triphala showed significantly lower mean gingival and plaque index scores at follow up of 2 weeks than baseline (p<0.001). There was no significant change in the mean gingival index between two groups (p=0.826). However the percentage change in plaque index was significantly higher in the chlorhexidine group (p=0.048) [35]. A crossover study which assessed the antiplaque and anti-gingivitis efficacy of triphala among school children, both triphala and chlorhexidine yielded a significant reduction in plaque and gingival index scores as compared to negative control (p<0.001). However there was no significant difference between the scores obtained between chlorhexidine and triphala mouthwashes at 3 phases of 1-month duration and a washout period of 15 days [37]. The study conducted among the age group of 25 to 40 years where they compared the triphala mouthwash with that of the chlorhexidine and placebo, the results showed that there was no significant difference between triphala and chlorhexidine mouthwash in terms of plaque index, gingival index and OHI-S from baseline to 7,30 and 60 days follow up [33]. When triphala and ela decoction were compared with 0.2% chlorhexidine mouthwash among age group of more than 18 years, the study results at 14th day showed that the reduction of plaque index from baseline for Triphala and Ela decoction was 42.59% and for Chlorhexidine it was 38.62% while at 2st day the reduction was 56.20% and 68.57% respectively. In comparison with the gingival index for Triphala and Ela decoction with Chlorhexidine mouthwash the reduction from baseline to 14 days was 31.95% and 38.62% respectively while from baseline to 21 days was 69.95% and 68.57% respectively [38]. Out of 4 studies on green tea mouthwash, the study evaluated the antiplaque efficacy of green tea catechin mouthwash with chlorhexidine mouthwash in the age group of 18-25 years; the results showed that difference between plaque score were not statistically significant (p>0.05) [39]. Also the results showed that both green tea and chlorhexidine mouthwash had comparable results in plaque reduction. Whereas another study reported that there was significant in-group difference in plaque and gingival indices after 1 and 4 weeks compared to baseline [34]. The study which evaluated the commercially available green tea mouthwash with Mint and saline rinse among the age group of 30 to 45 years at baseline, 2 weeks, 3 weeks and 4 weeks after SRP showed that there was significant reduction in plaque, gingival scores in both groups but to greater extent in patients who used green tea mouthwash for one month [40]. Another study evaluated the antiplaque, anti gingivitis efficacy of 2% Green tea among the age group of 18-60 years, the results show that there was significant (p<0.05) reduction in mean gingival and plaque index score among the green tea group from baseline to 28 days. A statistically significant reduction (p<0.05) was found in the mean difference in GI scores in the green tea group (0.67±0.22) as compared to the placebo control and a statistically significant reduction (p<0.05) was observed in the mean difference in PI score in the green tea group (1.65±0.68) compared to the control group [41]. In 2 studies that evaluated neem mouthwash, the first study compared the effectiveness of 2% neem, 0.5% tea and 0.2% chlorhexidine, the study results showed that mean plaque and gingival scores were reduced over the 2-week trial period. Neem and tea showed better antiplaque and antigingivitis effects than chlorhexidine (p<0.05) [42]. Recent study evaluated the impact of neem containing mouthwash against plaque and gingival score; the study consisted of two phases as crossover study and with 1-week washout period, the result showed slight reduction in plaque level in the first phase as well as in the second phase. When comparison was made between chlorhexidine and neem mouthwash there was no significant difference. Both neem and chlorhexidine mouth wash showed reduction in gingival index scores in the first phase and there was a statistically significant difference in neem and chlorhexidine mouthwash groups at baseline and after intervention (0.005 and 0.01 respectively). After the washout period during the crossover, gingival index scores were reduced in both neem and chlorhexidine, but there was a statistically significant difference between the groups only at baseline scores (0.01) [43]. Out of 4 studies on the other antioxidant mouthwashes like polyherbal, turmeric, tea tree oil, study on turmeric mouthwash showed statistically significant reduction (p<0.05) in mean plaque index with chlorhexidine gluconate mouthwash when compared with turmeric mouthwash [44]. No significant difference in mean gingival index was seen when chlorhexidine mouthwash was compared with turmeric mouthwash at baseline to 14 and 21 days. Study on polyherbal mouthwashes showed that there was significant improvement in gingival and plaque scores from baseline to 14 days of trial in both polyherbal and chlorhexidine mouthwash [45]. However, there was no significant difference between polyherbal and chlorhexidine groups neither at day 7 nor day 14 of the trial. Study on aloe vera and tea tree oil showed statistically significant decrease in all plaque and gingival index score was noted after the use of both herbal preparations at the end of 4 weeks, which was maintained after the 2-week washout period (p<0.001) [46]. However the difference in plaque and gingival index scores between the group using aloe vera, tea tree oil and chlorhexidine, was not statistically significant. Whereas study on guava mouthwash showed that mean plaque index and gingival index scores from baseline to 30 days had notable changes between chlorhexidine and guava mouth rinse compared to placebo [47]. In 14 studies, 4 studies assessed the total microbial count on administration of antioxidant mouthwashes, one study specifically evaluated the streptococcus mutans level at the baseline and postoperatively, all the 4 studies showed that the microbial counts where reduced on the usage of antioxidant mouth compared to the baseline value, however they had similar microbiological effect has that of the chlorhexidine mouthwash [33,44,46, 47]. Quality assessment of 14 selected studies was carried out based on CONSORT Guidelines 4 major criteria to evaluate risk of bias: (1) Randomization, (2) Allocation concealment, (3) Assessor blinding and (4) Dropouts. It was considered low risk if 3 out of 4 criteria was satisfied, moderate risk if only 2 criteria was satisfied and high risk if none of the criteria was satisfied. In the present study, 11 studies had low risk of bias, 2 studies had moderate risk of bias and one had high risk of bias. In 4 studies of triphala mouthwash 2 studies had low risk whereas other two studies had moderate risk. In 4 studies of green tea mouthwash one study had high risk of bias, whereas other 3 studies had low risk of bias as shown in Table 2(see PDF). The level of evidence of the 14 selected studies were assessed based on Oxford Centre for Evidence-Based Medicine 12 studies had level II evidence, whereas 2 studies had level III of evidence.

## Discussion:

Periodontal tissue depends on natural antioxidants to overcome the oxidative stress and maintain homeostasis. When antioxidants are depleted, the ability of the periodontal tissue to overcome oxidative stress, maintain normal tissue and control bacterial damage appears to be compromised. Thus, to overcome oxidative stress natural antioxidants and recently the antioxidant mouthwashes are used in the treatment of periodontal disease. The literature evidence supports the hypothesis about the association pathway between the antioxidant defense and improved periodontitis. This association needs support and the establishment of causal relationships by evidence based clinical decisions regarding the use of antioxidants mouthwashes [27]. Thus we carried systematic review with the contemporary methodological principles to reflect the highest available evidence in this approach. The qualitative synthesis of the present systematic review compared antioxidant mouthwashes i.e., Triphala, Green tea extract, Neem, Turmeric, Guava leaf extract, Polyherbal (Zingiber officinale, Rosmarinus officinalis and Calendula officinalis), aloe vera and tea tree oil to either placebo, Chlorhexidine mouthwash or Mint mouthwash. Most of the included studies showed similar antiplaque and antigingivitis effect as that of the chlorhexidine where as one study on neem mouthwash had better antiplaque and antigingivitis effect than that of chlorhexidine. Within the included studies in the systematic review 4 studies assessed the microbiological parameter and the results showed that the microbial counts where reduced on the usage of antioxidant mouth, however they had similar microbiological effect as that of the chlorhexidine mouthwash [33, [44,46,47]. Triphala showed similar antiplaque and antigingivitis effects as that of chlorhexidine in all the four studies. Triphala is an equiproportional mixture of Terminalia chebula, Terminalia bellerica and Emblicusofficialis. It has been used as a potent anti-inflammatory, antioxidant [48] and antimicrobial agent against a wide spectrum of microbes [49]. Studies have stated that the presence of tannins in triphala could effectively reduce the number of bacteria available for binding to the tooth surface by increasing their physical removal from oral cavity through aggregate formation [49]. The effective inhibition of glucosyl transferase activity and reduced bacterial adhesion to hydroxyapatite, as seen with the presence of tannin extracts, suggests antiplaque activity. Triphala also exhibits a strong antioxidant property, E.officinalis present in triphala contains enormous amount of ascorbic acid that act as a chain breaking antioxidant and impairs the free radicals throughout the body, which might help in significant reduction in gingival score when triphala mouthwashes are used.

Studies have showed that there is no significant difference in the effectiveness between triphala mouthwash and the gold standard chlorhexidine from which they concluded that the antiplaque and antigingivitis activity of triphala is similar to that of chlorhexidine [37]. Triphala mouth rinse has broad antibacterial action against Gram-positive-negative microorganisms. It exhibits antioxidant activity, as well as strong inhibitory activity on polymorphonuclear leukocyte-type matrix metalloproteinase involved in the extracellular matrix degradation during periodontitis. Thus triphala acts as an effective, economic alternative in reducing plaque and gingivitis. It can be used in short-term treatment regimens without potential side effects of chlorhexidine [35]. Triphala mouthwash was found to decrease the inflammatory parameters and thus, leading to improvement in gingivitis and concluded that triphala mouthwash can be considered a potential therapeutic agent in treatment of gingivitis [33]. Triphala combined with Ela decoction had similar efficacy as that of chlorhexidine in reducing biofilm build up and in treatment of gingivitis [38]. All the 4 studies on green tea mouthwashes showed antiplaque and antigingivitis activity similar to that of chlorhexidine mouthwash.The antiplaque and antigingivitis activity of green tea could be because of polyphenols that are well known for their antioxidative activity, providing protection against degenerative diseases and acting as antitumorigenic agents. Green tea extract has been shown to be effective against oxidative stress by establishing the balance between antioxidants and reactive oxygen within the cells. Green tea mouthwash acts are as effective as chlorhexidine against plaque regrowth and gingival inflammation with less tooth staining [34]. Studies suggested that there is a significant reduction seen in the plaque, gingival and periodontal scores with green tea and mint mouth washes but green tea mouthwash proved to be more beneficial than mint mouthwash [40]. Green tea mouthwash is beneficial to cure or prevent periodontal disease due to the presence of catechin and epigallocatechin; these derivatives inhibit various periodontal pathogens. Studies demonstrated a decrease in the plaque and gingival scores in the subjects using a green tea mouth rinse, showing the antiplaque and anti-gingivitis effect of green tea [41]. Studies have indicated that green tea catechin mouthwash has a comparable antiplaque efficacy to chlorhexidine gluconate when used for a period of 7 days [39]. Green tea catechin mouthwash due to its better taste and no known side effects can be used on a daily basis as an alternative for chlorhexidine gluconate as an anti-plaque agent.

Out of the two studies included 1 study showed that neem mouthwash had better antiplaque and anti-gingivitis activity than chlorhexidine mouthwash whereas the other study showed that neem mouthwash had similar effect as that of chlorhexidine. As such neem contains trimethylamine, chlorides, nimbidin, azadirachtin, lectin, fluorides in large amounts and silica, sulfur, vitamin C, tannins, saponins, flavonoids, and sterols in small quantities. Neem as a mouthwash is effective on both Gram-positive and Gram-negative organisms. Its antiplaque and anti-gingivitis effect could be because neem leaf extract, contains polyphenols that adhere to oral surfaces that had shown to provide long-lasting antibacterial as well as synergic antioxidant activities when in complex with bacteria, red blood cells and lysozyme which makes to be effective in periodontal disease [50]. Studies have assessed the antiplaque and anti-gingivitis activities using 2% neem and their results that when neem used at 2% concentration had antiplaque and anti-gingivitis activity compared to that of the gold standard chlorhexidine [42]. However, some study showed that 2% neem had antiplaque and anti-gingivitis activities where similar and comparable to that of the chlorhexidine, the variation could be due to shorter follow up period and since there was washout period in this study [43]. A recent systematic review shows the similar results as obtained in this systematic review where they showed the neem mouth rinse was as effective as chlorhexidine mouthrinse when used as an adjunct to tooth brushing in reducing plaque and gingival inflammation in gingivitis patient [51].

Numerous other antioxidants like guava leaf extract, polyherbal containing ginger, aloe vera and tea tree oil, turmeric studied by various authors [44-47] showed that these antioxidants have antiplaque and antigingivitis effects which makes them as an alternative herbal mouthwashes in treatment of periodontal diseases. However all these studies show that their antiplaque and antigingivitis effects were equal and comparable to that of the gold standard mouthwash but better when compared to the placebo mouthwashes. Thus, the present systematic review was undertaken to evaluate the efficacy of antioxidant mouthwashes in periodontal treatment. The results of the Quality assessment of 14 included studies based on CONSORT guidelines revealed that the majority 11 studies had low risk of bias, 2 had moderate risk of bias and one had high risk of bias. Between those 2 studies on triphala had low risk whereas other two had moderate risk. In 4 studies of green tea mouthwash one study had high risk of bias, whereas other 3 studies had low risk of bias. The level of evidence was assessed based on Oxford Centre for Evidence-Based Medicine in that 12 studies had level II evidence, whereas 2 studies had level III of evidence.

A systematic review involves the application of methodological strategies that limit bias and evaluate and summarize crucial scientific evidence. These systematic analyses can help practitioners be aware of the scientific literature [52]. The search strategy used in this study included the most important databases to health sciences in addition to the PICO's strategy, which allows the comparison of the clinical trial results, verifying if there is an additional effect in the use of antioxidant mouthwashes as adjuvants in conventional periodontal therapy. The search results showed that this approach has been slightly studied, especially in considering the antioxidant diversity, as well as the evaluated parameters. Nevertheless, knowing that the antioxidants used have common objectives, the results can be interpreted as a role of antioxidants mouthwashes in general as adjuvants to conventional periodontal therapy Based on the systematic review, antioxidant mouthwash seemed to be beneficial and had a comparative similar effect as that of gold standard chlorhexidine mouthwash. Even though we have included the major electronic databases like google scholar, pub-med, Cochrane the limitation would have been that other electronic database searches were not included. Clinical trials in relation to antioxidants mouthwashes are limited with moderate to high risk of bias. There is only one study in the current systematic review, which evaluated the side effects of the antioxidant mouthwashes. Though there are studies on antioxidant mouthwash, there are minimal studies evaluating the individual ingredients. All the studies included in the current systematic review had different postoperative intervention time which made it difficult to conduct Meta analysis based on the current available data. Thus, the future studies require comparing the individual antioxidants mouthwashes with chlorhexidine mouthwashes evaluating the long-term side effects and more homogeneous studies with low risk of bias are required to conduct meta-analysis in this aspect.

## Conclusion

We report gleaned clinical data on mouthwash with antioxidants having anti-gingivitis, antiplaque and antimicrobial effects similar to chlorhexidine.
